# Mediation Effect of CSF Substance P on the Association Between Smoking and Sleep

**DOI:** 10.1002/brb3.70296

**Published:** 2025-02-09

**Authors:** Xie Zhang, Lingling Chen, Yuyu Wu, Yu‐Hsin Chen, Xingguang Luo, Zeping Xu, Weiming Hu, Yimin Kang, Li Chen, Yanlong Liu, Fan Wang, Danhui Liu

**Affiliations:** ^1^ Department of Pharmacy The Affiliated Lihuili Hospital of Ningbo University Ningbo China; ^2^ School of Mental Health Wenzhou Medical University Wenzhou China; ^3^ Department of Psychiatry Yale University School of Medicine New Haven Connecticut USA; ^4^ Department of Psychiatry The Third Hospital of Quzhou Quzhou China; ^5^ Medical Neurobiology Lab Inner Mongolia Medical University Huhhot China; ^6^ Beijing Hui‐Long‐Guan Hospital Peking University Beijing China; ^7^ School of Laboratory Medicine and Life Sciences Wenzhou Medical University Wenzhou China

**Keywords:** cerebrospinal fluid substance P, cigarette smoking, mediation, Pittsburgh Sleep Quality Index, sleep disorders

## Abstract

**Background:**

Cigarette smoking has been linked to severe and persistent sleep disturbances alongside notable fluctuations in neuropeptide levels. Substance P (SubP), influenced by smoking, also impacts sleep‐wake cycles. However, its specific role in smoking‐induced sleep disorders remains unclear. This study aimed to explore the connection between cigarette smoking and sleep quality by examining SubP levels in cerebrospinal fluid (CSF) and identifying potential treatment avenues for sleep disorders.

**Methods:**

A total of 146 Chinese men (93 nonsmokers, 53 active smokers) undergoing lumbar puncture before anterior cruciate ligament reconstruction were enrolled. Clinical data and Pittsburgh Sleep Quality Index (PSQI) scores were assessed, followed by CSF sample collection and CSF SubP level measurement.

**Results:**

Active smokers exhibited significantly higher PSQI scores (4.02 ± 2.27 vs. 2.60 ± 2.46, *p* < 0.001) and CSF SubP levels (2111 ± 212 vs. 1821 ± 289, *p* < 0.001) compared to nonsmokers. A negative correlation (*r* = −0.434, *p* < 0.001) between SubP levels and PSQI scores was observed in all participants and nonsmokers, while no correlation (*r* = −0.044, *p* = 0.72) was found in active smokers. Logistic regression analysis across different dimensions of sleep disorders indicated associations between CSF SubP levels and sleep quality as well as daytime dysfunction (OR = 0.439 (0.211–0.891), *p* = 0.025; OR = 0.308 (0.152–0.608), *p* = 0.001). Mediation analysis suggested that CSF SubP levels mediated the relationship between smoking and sleep.

**Conclusion:**

CSF SubP levels are elevated in active smokers and appear to play a mediating role in the relationship between smoking and sleep regulation, as evidenced by a negative correlation between CSF SubP levels and PSQI scores.

## Graphical abstract

Cigarette smoking is associated with sleep disorders measured by PSQI. CSF SubP mediates the relationship between cigarette smoking and sleep disorders. This study provides insights into how cigarette smoke induces sleep disorders.

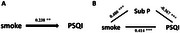

## 1 Introduction

Cigarette smoking poses a significant global health threat, causing harm to nearly every organ in the body. It stands as the primary culprit behind over 30% of all cancer‐related deaths, 80% of chronic obstructive pulmonary disease fatalities, and a substantial portion of premature cardiovascular mortality (G. B. D. T. Collaborators 2017). Beyond its direct impact, smoking indirectly contributes to various health issues, including physical and mental distress, by disrupting sleep patterns among smokers (Liao et al. 2019; Liu et al. 2020). Extensive evidence from population‐based, laboratory, and clinical studies underscores the negative correlation between cigarette smoking and sleep quality in both adults and adolescents (Catoire et al. 2021; Hwang and Park 2022; Liu et al. 2020). These studies consistently reveal that smokers experience compromised sleep efficiency, reduced total sleep duration, disruptions in slow‐wave sleep, increased sleep onset latency, and other indicators of diminished sleep quality compared to nonsmokers (Catoire et al. 2021; Liao et al. 2019; McNamara et al. 2014). Additionally, smoking is associated with an elevated risk of developing various sleep disorders, such as restless legs syndrome, sleep apnea, and alterations in sleep architecture, further exacerbating health risks (Hwang and Park 2022; Liao et al. 2019). These sleep disturbances can have profound repercussions on individuals' well‐being, highlighting the urgent need to understand the relationship between smoking and sleep disorders and explore effective treatment options.

Smoking‐induced sleep disturbances are primarily attributed to the pharmacological effects of nicotine on brain neurotransmission (Branstetter, Krebs, and Muscat 2022; Leonel et al. 2020). Nicotine's activation of cholinergic receptors leads to the release of neurotransmitters, including dopamine, norepinephrine, serotonin, acetylcholine, and substance P (SubP) (Smith, Dwoskin, and Pauly 2010), all crucial for regulating sleep and wakefulness. SubP, an 11‐amino‐acid neuropeptide, exerts influence over sleep physiology (Hecht et al. 1980). It colocalizes with neurotransmitters in cells and brain regions implicated in sleep regulation, such as serotonin within the raphe nucleus, dopamine in the midbrain and striatum, and adrenocorticotropin‐releasing hormone within the hypothalamus (Otsuka and Yoshioka 1993; Sergeyev, Hokfelt, and Hurd 1999). Functioning through binding to neurokinin (NK) receptors, primarily NK‐1R, which are abundant in the human brain (Caberlotto et al. 2003), SubP is widely distributed in regions crucial for sleep regulation within the central nervous system (CNS), including the hypothalamus, brainstem, and cortex (Brown et al. 2012; Dam, Escher, and Quirion 1988). Laboratory experiments demonstrate that SubP administration enhances slow‐wave activity in mice and increases nonrapid eye movement sleep in rats (G. Zhang et al. 2004; Zielinski et al. 2015). Furthermore, low CSF SubP levels have been associated with anxiety and depression, closely linked to sleep problems (Carpenter et al. 2008). Despite these findings, the precise role and underlying mechanism of SubP in the sleep‐wakefulness cycle remain incompletely understood.

Our previous research indicated an association between smoking and elevated SubP levels in the cerebrospinal fluid (CSF) of active smokers (Wang et al. 2021). However, to date, no study has explored the relationship between smoking, CSF SubP levels, and sleep quality. In this study, we utilized the Chinese version of the Pittsburgh Sleep Quality Index (PSQI) to assess sleep quality, aiming to delineate the interplay among smoking, CSF SubP levels, and sleep, with the ultimate goal of identifying potent therapeutic strategies for addressing sleep disturbances.

## 2 Materials and Methods

### 2.1 Participants

A total of 146 Chinese adult males participated in this study, comprising 53 active smokers and 93 nonsmokers. Demographic information, including age, years of education, and body mass index (BMI), was recorded. Clinical data, such as history of substance abuse and dependence, were obtained via self‐report and corroborated by next of kin and family members. Exclusion criteria encompassed: (1) a familial history of neurological or psychiatric disorders; (2) diagnoses of systemic or CNS diseases according to the Mini International Neuropsychiatric Interview. The nonsmoking cohort consisted solely of individuals who had never smoked and had no history of substance abuse or dependence. Active smokers were defined as those consuming at least half a pack of cigarettes daily (equivalent to 10 cigarettes) for over a year, following the *Diagnostic and Statistical Manual of Mental Disorders*, 4th Edition. Smokers consuming fewer than 10 cigarettes per day were excluded. Participants with a history of alcoholism or psychiatric disorders were not included. Approval for the study was obtained from the Institutional Review Board of Inner Mongolian Medical University (YKD2014031) on March 11, 2014, adhering to the Declaration of Helsinki, with written informed consent obtained from all participants.

### 2.2 Assessments, Biological Sample Collection, and Laboratory Tests

Sleep quality was assessed using the Chinese version of the PSQI (Tsai et al. 2005), a widely utilized scale comprising 19 items. Each item is rated on a 4‐point Likert scale (ranging from 0 to 3), contributing to seven sub‐scales: subjective sleep quality, sleep latency, sleep duration, habitual sleep efficiency, sleep disturbances, use of sleep medication, and daytime dysfunction. Total scores range from 0 to 21, with higher scores indicating poorer sleep quality.

Lumbar puncture is routinely performed as part of the standard clinical procedure for patients undergoing anterior cruciate ligament reconstructive surgery in China, facilitating CSF sample collection with minimal disease entity interference. Smoking cessation prior to this procedure was not mandated. A licensed anesthetist conducted lumbar punctures in the morning before surgery using 3 mL of 0.5% ropivacaine for local anesthesia, and 5 mL CSF samples were obtained via intrathecal collection. Samples were promptly frozen at −80°C. The anterior cruciate ligament reconstruction procedure typically lasted less than 1 hour, with participants hospitalized for a maximum of 2 days before surgery. SubP levels in CSF were quantified using commercial radioimmunoassay kits from Phoenix (Phoenix Pharmaceuticals, Inc., Burlingame, CA, USA), following the manufacturer's instructions, with laboratory technicians blinded to clinical data.

### 2.3 Statistical Analysis

The normality of all variables was assessed using the Shapiro–Wilk test. Age, BMI, and SubP demonstrated nonnormal distributions (*p* < 0.05). Consequently, the Mann–Whitney rank‐sum test and independent *t*‐test were employed to compare differences in general demographic data, clinical data, and raw biomarkers between groups. Spearman correlation analysis was conducted to evaluate the relationship between SubP and PSQI scores. Linear regression analyses were then conducted to explore the mediating effect of SubP on PSQI scores, with gender, age, BMI, marital status, and living arrangements included as control variables. A mediation analysis was performed to elucidate whether SubP mediated the relationship between smoking and PSQI scores. Logistic regression was utilized to elucidate the relationship between the seven subdomains of PSQI and SubP in all participants. All statistical analyses were conducted using R Programming Language 4.2.0 and the R package (Bruce R). All tests were two‐sided, with the significance threshold set at *p* < 0.05.

## 3 Results

### 3.1 Sociodemographic and Clinical Characteristics

Table 1 presents the results. Age (29 ± 9 vs. 33 ± 9, *p* = 0.013), BMI (24.8 ± 4.1 vs. 25.6 ± 2.8, *p* = 0.035), SubP (1821 ± 298 vs. 2111 ± 212, *p* < 0.001), PSQI global score (2.60 ± 2.46 vs. 4.02 ± 2.27, *p* < 0.001), sleep quality (0.51 ± 0.64 vs. 0.72 ± 0.60, *p* = 0.028), sleep latency (0.32 ± 0.61 vs. 0.72 ± 0.63, *p* < 0.001), and sleep disturbance (0.40 ± 0.57 vs. 0.87 ± 0.48, *p* < 0.001) were significantly higher in the smoker group compared to the nonsmoker group. No significant differences were observed in other variables. Active smokers exhibited a significantly lower marriage rate (*p* = 0.011) and a higher rate of living alone or with family (*p* = 0.002) compared to nonsmokers.

**TABLE 1:** Comparisons between nonsmokers and active smokers.

Variable	Nonsmokers	Active smokers	*p*
	(*n* = 93) Mean ± SD (Median, IQR)	(*n* = 53) Mean ± SD (Median, IQR)	
Age	29 (9)	32 (9)	0.013^*^
BMI	24.8 (4.1)	25.6 (2.8)	0.035^*^
SBP	130 (13)	126 (13)	0.3
DBP	75 (9)	76 (12)	0.9
SubP	1821 (289)	2111 (212)	<0.001^***^
PSQI Global Score	2.60(2.46)	4.02 (2.27)	<0.001^***^
PSQI component	No symptom/with symptom	No symptom/with symptom	
1.Sleep quality	53/40	19/34	0.014^*^
2.Sleep latency	69/24	19/34	<0.001^***^
3.Sleep duration	45/48	27/26	0.8
4.Sleep efficiency	86/7	47/6	0.5
5.Sleep disturbance	59/34	10/43	<0.001^***^
6.Sleep medication	89/4	52/1	0.7
7.Daytime dysfunction	52/41	24/29	0.2
Marriage			0.011^*^
Married	50 (54%)	17 (32%)	
Unmarried	43 (46%)	36 (68%)	
Living			0.002^**^
Alone or with family	68 (73%)	50 (94%)	
With others	25 (27%)	3 (5.7%)	

*Note*: Marital status was reported as the chi‐square test between nonsmokers and active smokers, and other data were reported as Mann–Whitney sum tests between nonsmokers and active smokers.

* *p* < 0.05.

** *p* < 0.01.

*** *p* < 0.001.

### 3.2 Correlation Between SubP Levels and PSQI Scores

**FIGURE 1:**
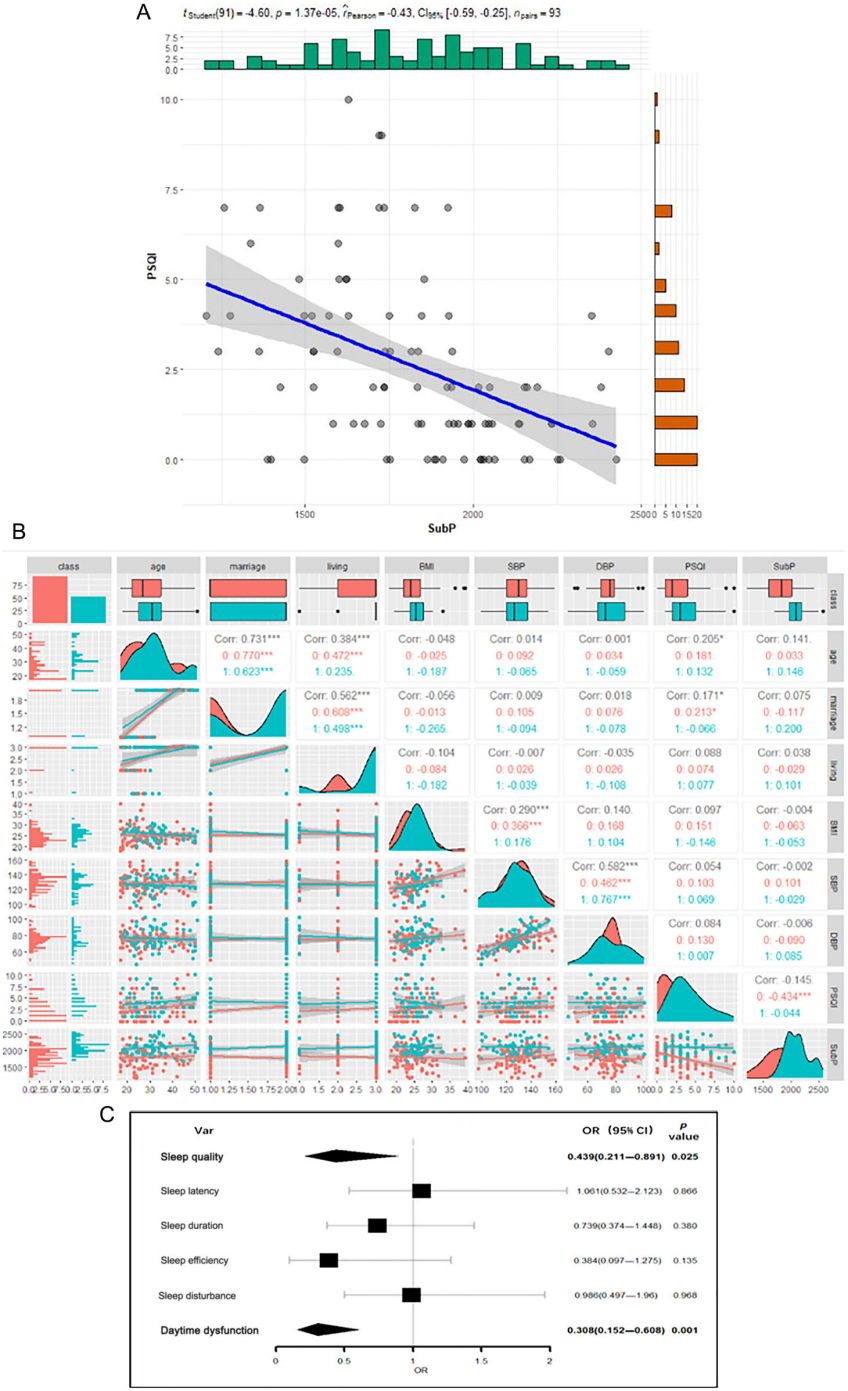
Correlation analysis between Substance P and PSQI scores. (A) Linear regression model was used to analyze the relationship between CSF SubP levels and PSQI scores in all participants. (B) Bivariate correlation matrix for the study variables in nonsmokers and active smokers using Spearman's rank correlation coefficients analysis. (C) SubP levels (divided into two groups by the median level) and six components of PSQI (yes/no) were included into the logistics regression model as dichotomous variables to assess the associations between the two. The linear regression and logistic regression models were adjusted for age, body mass index, marital status and living arrangement. Statistical significance (*p* < 0.05) was denoted in bold. *p < 0.05. CI, confidence interval; OR, odds ratio; PSQI, Pittsburgh Sleep Quality Index; SubP, Substance P.

A negative correlation was observed between SubP and PSQI scores in all participants (*r* = −0.145, *p* < 0.001, see Figure 1A). Spearman's correlations revealed a consistent relationship between CSF SubP level and PSQI scores in nonsmokers (*r* = −0.434***, *p* < 0.001). Additionally, logistic regression analysis demonstrated significant associations between PSQI subgroups (sleep quality and daytime dysfunction) and CSF SubP level, with adjustments for age, education, and BMI (all *p* < 0.05, see Figure 1C).

### 3.3 Mediation Analysis

**FIGURE 2:**
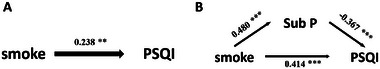
Effect decomposition of mediation models in PSQI scores. (A) Total effect between smoke and PSQI scores. (B) Effect decomposition of the mediation model for the relationship between smoke and PSQI scores association with CSF SubP levels as mediator. *p < 0.05, **p < 0.01, ***p < 0.001. PSQI, Pittsburgh Sleep Quality Index; SubP, Substance P.

To elucidate whether the relationship between smoking and PSQI scores is mediated by SubP, three separate mediation models were conducted. The mediation effect of SubP was confirmed in the association between smoking and PSQI scores (see Table 2 or 3 and Figure 2). Linear regression results showed a positive effect of smoking on PSQI scores (*β* = 0.238, *t* = 2.88, *p* < 0.01) after adjusting for age, BMI, marital status, and living arrangement. Similarly, a positive effect of smoking on CSF SubP level was observed. The addition of mediator variables demonstrated the mediating effect of CSF SubP level (*β* = −0.367, *t* = −4.259, *p* < 0.001).

**TABLE 2:** Linear regression table for the mediation analysis.

	Model1 (PSQI)		Model2 (SubP)		Model3 (PSQI)	
	*β*	*t*	*β*	*t*	*β*	*t*
Age	0.161	1.372	0.159	1.467	0.219	1.966
BMI	0.082	1.018	−0.052	−0.694	0.063	0.828
Marriage	−0.005	−0.03	−0.166	−1.363	−0.066	−0.524
Living	0.023	0.24	0.038	0.421	0.037	0.406
Smoke	0.238^**^	2.88	0.480^***^	6.297	0.414^***^	4.698
SubP	—	—	—	—	−0.367^***^	−4.259
*R* ^2^	0.076		0.210		0.177	
*F* (df)	3.386 (5140)		8.722 (5140)		6.19 (6139)	

*Note*: All data were reported as mediation analysis.

** *p* < 0.01.

*** *p* < 0.001.

**TABLE 3:** Effect decomposition from mediation models.

Effect decomposition	Estimated	95% CI		*p*
		Lower	Upper	
Indirect effect	−0.176	−0.267	−0.091	<0.001^***^
Direct effect	0.414	0.242	0.583	<0.001^***^
Total effect	0.238	0.060	0.406	0.006^**^

*Note*: All data were reported as mediation analysis. Model 1 was the linear regression model including smoke as the independent variable and PSQI as the dependent variable. Model 2 showed the linear regression between smoke and CSF SubP levels. Model 3: Both smoke and CSF SubP levels were included as factor to assess the effects of PSQI. All models were adjusted for age, BMI, marital status and living arrangement. All data were reported as mediation analysis.

Abbreviations: 95% CI, lower limit and upper limit of the 95% confidence interval; BMI, body mass index; PSQI, Pittsburgh Sleep Quality Index; SubP, Substance P.

** *p* < 0.01.

*** *p* < 0.001.

Bootstrap sampling revealed that smoking had a direct effect on PSQI scores (estimated = 0.414 (95% CI = 0.242–0.583), *p* < 0.001), as well as an indirect effect (estimated = −0.176 (95% CI = −0.267 to −0.091), *p* < 0.001), and total effect (estimated = 0.238 (95% CI = 0.060–0.406), *p* < 0.01). The mediating effect of CSF SubP level was found to be incomplete.

## 4 Discussion

In this study, we utilized human CSF SubP levels to examine the association between cigarette smoking and sleep quality. Our key finding reveals that smoking is indeed associated with impaired sleep quality, as evidenced by higher PSQI scores and elevated CSF SubP levels in active smokers compared to nonsmokers (see Table 1). These findings align with previous research demonstrating interactions between SubP and sleep disorders (Lieb et al. 2002; Ursavas et al. 2007; Zielinski et al. 2015), corroborating our observation of a negative correlation between CSF SubP levels and PSQI scores across all subjects. Furthermore, logistic regression analysis identified two PSQI subgroups (sleep quality and daytime dysfunction) significantly correlated with CSF SubP levels. Moreover, mediation analysis indicated that CSF SubP levels mediated the relationship between smoking and sleep quality, as measured by PSQI scores.

It is widely recognized that smoking significantly impacts sleep quality across various cohorts. Numerous studies have reported that smokers exhibit primary symptoms of insomnia, including decreased overall sleep duration, reduced sleep efficiency, prolonged sleep latency, diminished slow‐wave sleep, and increased daytime sleepiness (Cohrs et al. 2014; Jaehne et al. 2009; L. Zhang et al. 2006). Our findings align with these observations, demonstrating a correlation between smoking and higher PSQI scores indicative of poorer sleep quality. Nicotine in cigarettes can modulate sleep‐wake cycles by activating nicotinic receptors in crucial cholinergic circuits (Htoo et al. 2004) and dopaminergic neurons within the ventral tegmental area (Picciotto et al. 1998). Additionally, our study revealed significantly elevated CSF SubP levels in active smokers compared to nonsmokers (2111 ± 212 vs. 1821 ± 289, *p* < 0.001, see Table 1), consistent with our previous research (Wang et al. 2021). Previous studies have reported that chronic exposure to cigarette smoke upregulates SubP expression in CNS neurons (Canning and Spina 2009; De Swert et al. 2009). This phenomenon may be attributed to the inactivation of neutral endopeptidase by cigarette smoking, leading to reduced SubP degradation and subsequent elevation of SubP levels (Wong et al. 2004).

Additionally, our secondary finding suggests a potential negative correlation between CSF SubP levels and sleep quality assessed by PSQI scores in all participants (see Figure 1A), with sleep quality and daytime dysfunction emerging as two subgroups revealing significant differences (see Figure 1C). SubP is a well‐studied neuropeptide belonging to the tachykinins family and is widely distributed throughout the nervous system (Hidese et al. 2023). In the CNS, SubP is prominently found in regions such as the midbrain periaqueductal gray, nucleus raphe magnus, nucleus reticularis gigantocellularis pars α, posterior hypothalamus, basal forebrain, basal ganglia, nucleus accumbens, and cerebral cortex (Bright, Vink, and Byard 2018; Kaczyńska et al. 2018; Zieglgänsberger 2019). Consequently, SubP is intricately involved in regulating various physiological and pathophysiological processes, including sleep‐wake states (Bright, Vink, and Byard 2018; Kaczyńska et al. 2018; Zieglgänsberger 2019). Notably, the NK‐1 receptor (NK‐1R), to which SubP binds, is distributed widely across the brain, including areas crucially involved in sleep regulation, such as the hypothalamus, brainstem, and cortex (Brown et al. 2012; Dam, Escher, and Quirion 1988).

Several studies have reported the sleep‐promoting effects of SubP. For instance, research has shown a significant positive correlation between serum levels of SubP and slow‐wave sleep (Ursavas et al. 2007). Furthermore, studies utilizing NK‐1R antagonists have demonstrated an association between reduced SubP levels and augmented sleep (Kramer et al. 1998). Microinjection of SubP into the bilateral ventrolateral preoptic area in rats has been shown to enhance slow‐wave sleep (G. Zhang et al. 2004), while injection of SubP into the cortex of mice has potentiated slow‐wave activity (Zielinski et al. 2015). Additionally, SubP conjugated with cholera toxin A subunit has been found to increase nonrapid eye movement sleep slow‐wave activity (Zielinski and Gerashchenko 2017).

It is evident from our findings that smoking is associated with higher PSQI scores and CSF SubP levels, while higher CSF SubP levels are linked to lower PSQI scores. This raises the question: What is the actual relationship and interaction among smoking, CSF SubP levels, and sleep quality assessed by PSQI scores? Our results suggest that SubP might serve as a mediator in the process of smoking affecting PSQI scores (see Table 2 or 3 and Figure 2). One potential mechanism underlying SubP's mediation between smoking and sleep involves the influence of smoking on NK‐1R expression in the human brain. In the cerebral cortex, NK‐1R is exclusively expressed in type I nitric oxide synthase (nNOS) cells (Williams et al. 2019). Notably, type I nNOS cells play a pivotal role in linking homeostatic sleep drive with slow‐wave sleep (Dittrich et al. 2012) and have been identified as sleep‐active in mice (Pasumarthi, Gerashchenko, and Kilduff 2010). Moreover, research indicates that repeated and long‐term nicotine intake may elicit an augmentation in the synthesis or a diminution in the release of SubP via counteradaptations in opponent‐process dynamics (Pittenger et al. 2016). Likewise, smoking can up‐regulate NK‐1R expression in monocytes of healthy individuals (Amoruso et al. 2015). Thus, smoking may increase CSF SubP levels and NK‐1R expression, thereby activating type I nNOS cells, which in turn induce sleep.

In summary, our study suggests that cigarette smoking might potentially enhance SubP receptors, particularly NK‐1R, to influence sleep. However, the precise mechanisms underlying how smoking affects sleep, despite the mediation effect of CSF SubP levels, remain incompletely elucidated. Therefore, further investigation is necessary to better understand the mechanisms by which SubP modulates sleep‐wake behaviors in the context of smoking.

Several limitations in our study should be noted. Firstly, our reliance on subjective measures of sleep rather than objective assessments imposes certain constraints on the findings. Secondly, while CSF tissue analysis provides insights into biochemical changes, it does not directly assess neuronal functions in the brain. Thirdly, our study included patients with anterior cruciate ligament injuries rather than healthy individuals, potentially introducing confounding factors such as surgical stress that could affect biomarkers and sleep quality. Furthermore, the use of a cross‐sectional research design presents a fundamental methodological challenge, hindering the ability to derive causal inferences. Lastly, SubP seems to have a bidirectional function when it comes to regulating sleep (Shen et al. 2022), so there is a clear need for delving deeper into the mechanism of SubP mediating sleep in smokers and a larger sample size to effectively validate our results.

## 5 Conclusions

In conclusion, our study highlights the association between cigarette smoking, sleep disorders, and elevated SubP levels in the CSF of smokers compared to nonsmokers. Furthermore, SubP appears to play a mediating role in the relationship between smoking and sleep regulation, as evidenced by a negative correlation between CSF SubP levels and PSQI scores.

## Author Contributions

**Xie Zhang**: writing–original draft. **Lingling Chen**: writing–original draft. **Yuyu Wu**: writing–original draft, data curation, formal analysis. **Yu‐Hsin Chen**: writing–original draft, methodology, project administration. **Xingguang Luo**: writing–review and editing. **Zeping Xu**: investigation. **Weiming Hu**: project administration, supervision. **Yimin Kang**: methodology, project administration, supervision. **Li Chen**: writing–original draft, methodology, project administration. **Yanlong Liu**: writing–review and editing, supervision, conceptualization. **Fan Wang**: writing–review and editing, resources. **Danhui Liu**: writing–review and editing, supervision.

## Ethics Statement

Our study received ethical approval from the Institutional Review Board of Inner Mongolian Medical University (YKD2014031) on March 11, 2014. All participants provided their written informed consent to participate in this study and the consents were obtained from minors in addition to parental/guardian consent.

## Conflicts of Interest

The authors declare no conflicts of interest.

### Peer Review

The peer review history for this article is available at https://publons.com/publon/10.1002/brb3.70296.

## Data Availability

The data used to support the findings of this study are available from the published literature, further inquiries can be directed to the corresponding author.
